# Adiponectin in Periodontitis: A Narrative Review of Biology, Human Evidence, Mechanistic Models and Translational Perspectives

**DOI:** 10.3390/biology15100746

**Published:** 2026-05-08

**Authors:** Martyna Mochol, Włodzimierz Dura, Maike Lodigkeit, Piotr Andrzejewski, Mariusz Lipski, Małgorzata Mazurek-Mochol

**Affiliations:** 1 Department of Periodontology, Pomeranian Medical University in Szczecin, Powstańców Wlkp 72, 70-111 Szczecin, Poland; 2 Department of Preclinical Conservative Dentistry and Preclinical Endodontics, Pomeranian Medical University in Szczecin, Powstańców Wlkp 72, 70-111 Szczecin, Poland

**Keywords:** adiponectin, periodontitis, adipokines, obesity, type 2 diabetes mellitus, AdipoR1, AdipoR2, inflammation, alveolar bone loss

## Abstract

Periodontitis is a chronic inflammatory disease which is initiated by dysbiotic biofilms and maintained by a host who is permissive to inflammation resulting in continuous destruction of periodontal supporting structures. Periodontitis occurs frequently with obesity and type 2 diabetes mellitus and the broader cardiometabolic risk state leading to investigations into the common immunometabolic pathways that link these conditions. Adiponectin, an insulin sensitizing and anti-inflammatory adipokine which can also act as a vasculoprotective and bone-related factor, has been studied as a potential modulator of the relation between periodontal inflammation and systemic metabolic disturbance. This narrative review summarizes the biology of adiponectin and its receptors, human findings relating to both the local and circulating forms of adiponectin in periodontal health and disease, the mechanism in cell and animal models and translational implications and limitations. The literature was reviewed in a narrative manner with particular attention to study quality, compartment-specific biology and any conflicts in evidence and the difference between biological plausibility and clinical relevance. A tendency for a reduction in the circulating, saliva and gingival crevicular fluid levels of adiponectin in periodontitis in human studies, particularly those with co-existing obesity and type 2 diabetes mellitus, can be demonstrated but these finding are often disparate due to variable methods in case definitions, assay techniques, metabolic background of subjects and other confounders. Experimental findings may establish biological plausibility by linking adiponectin signalling with the mechanisms which affect inflammatory responses, endothelial function and matrix homeostasis, osteoclastogenesis and subsequent alveolar bone loss, although adiponectin signalling appears context-specific in its actions and this does not confirm clinical relevance. Evidence suggests adiponectin is a biologically significant, but context-dependent factor within the immunometabolic network which connects periodontal disease with the systemic condition, rather than a sole marker or clinically recognized target for therapeutic intervention.

## Simple Summary

Periodontitis is a chronic inflammatory disease that occurs when susceptible hosts encounter dysbiotic dental plaque and subsequent loss of support to the teeth. It is commonly associated with obesity, type 2 diabetes mellitus and other cardiometabolic risk factors, which may influence the hosts inflammatory response and disease progression. Adiponectin is an adipokine that is secreted from adipose tissue, has the role of regulating insulin sensitivity, inflammation, vascular endothelial function and bone metabolism. This narrative review aims to critically evaluate the adiponectin biology, signalling and human, cellular and animal studies with regard to periodontitis. A meta-analysis of hu-man studies suggests that, especially in the obese and type 2 diabetic periodontal patients circulating and local adiponectin levels are commonly reduced in periodontitis; however, these studies are not entirely concordant and study design, definition of disease, sampling technique, technology for adiponectin analysis and presence of major confounding factors vary among them. Causation can be inferred from laboratory studies in that adiponectin signalling can have some influence on inflammation, matrix, osteoclastogenesis and bone loss pathways; however the influence is context- and dose-dependent and does not trans-late to clinical evidence. While adiponectin appears to be an important component of the immunometabolic pathways between periodontitis and metabolic disease, current evidence suggests that it is neither a reliable marker for periodontitis nor a target to be manipulated at a therapeutic level.

## 1. Introduction

Periodontitis is a chronic inflammatory disease of the tooth-supporting tissues which is initiated by dysbiotic biofilms and mediated by a susceptible host response leading to clinical attachment loss, destruction of alveolar bone and, in advanced stages, tooth loss [1,2]. Whilst microbial dysbiosis remain the cardinal etiologic factor, the severity and progression of disease is regulated by host factors including immunomodulation, metabolic state, smoking and general systemic comorbidity [1,2,3]. In recent years, periodontitis has been studied as part of a broader multi-morbidity network encompassing obesity, type 2 diabetes mellitus and cardiovascular risk not because such conditions directly cause periodontitis but because they may influence the host inflammatory and metabolic environment in which periodontal destruction develops [2,3,4,5,6].

Adipose tissue is now regarded as an endocrine organ which releases adipokines capable of regulating aspects of immunity, vascular biology, glucose metabolism and bone remodelling [2,3,4]. Adiponectin within the adipokine network has received specific attention because, unlike most adipokines, it is generally considered to possess insulin sensitizing, endothelial protective and pro-inflammatory/pro-resolving signalling capabilities under many physiological and pathological conditions [1,2]. Adiponectin biology is, however, not consistent across tissues and disease states, and the concentration, isoform distribution, receptor binding and downstream effects may differ with obesity, glycemic status, inflammatory burden, sex, age and anatomical location of the specific tissue compartment, precluding a simple interpretation of adiponectin within the context of periodontitis.

To date several reviews and meta-analyses on adipokines, obesity and periodontitis in general have been published [1,2,3,4,5,6]. However, it is now appropriate to provide a specific review focused on adiponectin for the following reasons. First, current studies of serum, salivary, gingival crevicular fluid and tissue-associated adiponectin have been found to not always be consistent and such different compartments should not be interpreted as having equivalent physiological or metabolic effects. Second, much of the new work performed is not descriptive associations but examines receptor mechanisms, compartment-specific signalling and experiments using adiponectin receptor agonists preclinically. Third, there is currently considerable uncertainty in the translational interpretation of the research literature, a differentiation between evidence at human level and pre-clinical mechanistic investigations being particularly crucial.

Within the above framework, it is not the objective of this narrative review to treat adiponectin as a key driver of periodontitis, but rather as a context-dependent immunometabolic regulator capable of modulating host responses at the perio-systemic interface. We therefore critically assess the biological properties of adiponectin and its receptors, and review the data obtained from human studies of serum and local adiponectin in health and periodontitis, from both preclinical and clinical model of cell and animal studies, along with an evaluation of the strengths, limitations and translational implications of the research. The emphasis is specifically on the quality of human data and the distinction between the biological rationale and translational implications of adiponectin research.

In view of this backdrop, the present sections first evaluate the general biology and receptor signalling of adiponectin, followed by an examination of human data, experimental model research and clinical translation.

### Approach to the Literature for This Narrative Review

The aim of this article was to write a critical narrative review on adiponectin in periodontitis, focusing on its biological mechanisms, evidence from human observational and intervention studies, as well as mechanisms revealed by experimental evidence. The literature search was mainly based on MEDLINE/PubMed, Scopus and Google Scholar databases. It was conducted repeatedly during the whole writing process and was last updated in early 2026. Keywords used were combinations of adiponectin, periodontitis, periodontal disease, adipokine, obesity, type 2 diabetes, gingival crevicular fluid, saliva, AdipoR1, AdipoR2, AdipoRon and AdipoAI. Additional publications of interest were obtained by manual selection of reference lists of important original papers, narrative reviews, systematic reviews and meta-analyses.

Priority was given to English-language original studies which directly explore adiponectin biology in periodontal health and disease: human clinical studies on plasma or local levels of adiponectin; studies on expression of adiponectin receptors and signal transduction pathways in periodontal and oral tissues; clearly mechanistically relevant in vitro and in vivo studies; and relevant reviews to put the biology or clinical picture in context. When an issue was addressed both in an original paper and in a subsequent review or meta-analysis, the original paper was preferred when dealing with a specific result and a review or meta-analysis was preferentially used to provide an overarching context.

Due to heterogeneity of published literature in the experimental design, periodontal condition definition, systemic status, biological sample origin, and laboratory technique, the review was designed not as a formal systematic review, as it did not aim at quantitative meta-analysis or formal risk of bias assessment. Instead, evidence was critically evaluated with an emphasis on the four issues: which findings are widely agreed upon; where are the discrepancies within literature, models or tissues; which hypotheses have evidence supporting them in humans; and which findings are still predominantly mechanistic/preclinical. The review was constructed on such an approach to achieve a clinically and biologically focused synopsis and avoid a misleading perception of absolute certainty.

## 2. Biology of Adiponectin and Its Receptors

### 2.1. Adiponectin Structure, Isoforms and Circulating Pools

Adiponectin is a 30 kDa adipokine secreted primarily by white adipocytes and it is classified as belonging to the C1q/tumour necrosis factor super-family [1,7]. It contains a collagen-like N-terminal globular portion with an affinity for receptors. It is present not as a single molecule, but in various complexes of varying size (molecular weights from 70 kDa to >1 MDa). The major components that appear in plasma are low-molecular-weight trimers, medium-molecular-weight hexamers and high-molecular-weight (HMW) multimers consisting of 12 or more monomers [1,7]. Full-length adiponectin can be cleaved by a protease to form globular adiponectin which appears to have a different receptor affinity and may be the cause of some differences seen between tissues.

Structural variations between adiponectin isoforms are relevant as the forms appear to be functionally non-equivalent. HMW adiponectin has often been described as the fraction most associated with enhanced insulin sensitivity, protection against endothelial damage and with some anti-inflammatory actions [1,7]. Thus, ratio of HMW to total adiponectin may carry information that is not represented by total adiponectin alone and a decreased ratio has been associated with poorer periodontal status in some cohorts [8]. This distinction has significance when evaluating the literature of periodontal disease as different studies may be referencing either the ratio, total adiponectin or the HMW portion and thus may not be referring to the same biological effect.

Circulating levels of adiponectin are often high in comparison with other cytokines, typically around 5–30 g/mL in healthy individuals [1,7]. However, basal levels do fluctuate according to gender, age, fat distribution and metabolic phenotype. Females generally demonstrate higher concentrations of adiponectin than males, and higher concentrations also exist in individuals where subcutaneous fat is more pronounced than visceral fat [1,2,5,7,8]. These physiologically apparent differences do not necessarily conflict with disease-related findings, but they demonstrate that the effect on plasma concentration is influenced by a complex set of endocrine factors. Lower levels of expression have also been identified in cells such as cardiac muscle, skeletal muscle, and some immune cells, possibly indicating paracrine production of adiponectin at sites other than adipocytes [1,7,9].

Adiponectin production and multimerization is known to be downregulated in obese subjects, insulin resistant subjects, and in response to chronic inflammation and the smoking habit. The metabolic phenotype also seems important [2,5,6,7,9]. Individuals with visceral obesity generally show lower circulating concentrations of adiponectin and a poorer HMW/total ratio. Obesity and loss of weight generally correlate with increased and decreased concentrations of adiponectin, respectively, and insulin sensitizers have been reported to increase concentrations [1,2,7]. This background is extremely relevant in the literature concerning periodontal disease, as a large proportion of subjects included within periodontal cohorts have been shown to be obese or have type 2 diabetes mellitus, which are conditions with a potentially significant impact on adiponectin concentrations independently of the periodontal condition itself.

### 2.2. Adiponectin Receptors and Signalling Pathways

Adiponectin primarily signals through two seven-transmembrane receptors AdipoR1 and AdipoR2 in conjunction with T-cadherin [1,7]. AdipoR1 expression is highest in skeletal muscle, though present also in immune and stromal cells, while AdipoR2 is expressed in the liver and many other tissues [1,7]. Unlike conventional G protein-coupled receptors, AdipoR1 and AdipoR2 have opposing membrane topology, with the N-termini on the intracellular face and the C-termini on the extracellular face and are functionally coupled to an inherent ceramidase-related activity [7]. AdipoR1 signalling is often associated with AMP-activated protein kinase (AMPK) dominated signalling while AdipoR2 is usually more associated with peroxisome proliferator-activated receptor alpha (PPAR)-dependent metabolic signalling, though there is considerable crossover between these systems [1,7]. T-cadherin is preferentially bound by the high molecular weight and hexameric forms of adiponectin and seems to predominantly facilitate adiponectin storage, tissue distribution and vasculoprotective effects rather than canonical intracellular signalling [7,9].

In the context of periodontitis, these receptors are highly relevant as AdipoR1 and AdipoR2 are present in the gingival tissues and in periodontal ligament cells [10,11,12,13] therefore forming the structural basis for local responsiveness to adiponectin. The expression of these receptors in these periodontal tissues alone does not confirm that adiponectin is protective uniformly; receptor expression varies with inflammation, and downstream effects may be specific to tissue compartment, adiponectin form, metabolic state and inflammatory signals.

Downstream signalling mechanisms involve adaptor proteins such as APPL1 and intersect with metabolic, oxidative, inflammatory and cell survival pathways [1,7]. The most central mechanism in AdipoR1-mediated effects is through AMPK activation which affects fatty-acid oxidation, mitochondrial function and insulin sensitivity [1]. AdipoR2-mediated effects have been shown to have additional intersect with transcriptional pathways related to PPAR [1,7]. Adiponectin signalling also includes activation of nuclear factor kappa B (NF-B), c-Jun N-terminal kinase (JNK), endothelial nitric oxide synthase and ceramide-related pathways [1,2,7,9,14]. These mechanisms could explain on a cellular level the observed influences of adiponectin on inflammation, vascularity and bony parameters.

**Figure 1:**
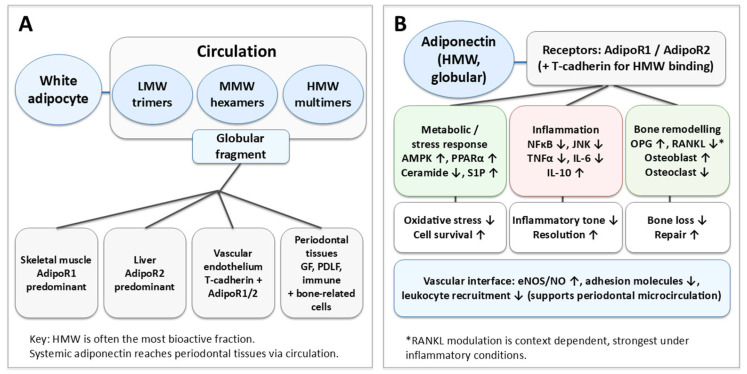
Adiponectin isoforms, receptors, and major signalling pathways relevant to periodontal inflammation and bone remodelling. Panel (**A**) illustrates the principal sources and circulating forms of adiponectin, including total adiponectin and its major multimeric forms, and the expression of adiponectin-related receptors in selected target tissues, including oral and periodontal tissues. Panel (**B**) summarizes the main signalling pathways linked to adiponectin receptor activation, including AMP-activated protein kinase (AMPK)-related signalling, peroxisome proliferator-activated receptor alpha (PPARα)-associated pathways, and downstream effects relevant to inflammatory regulation, endothelial function, and bone remodelling. In the periodontal context, these pathways are presented as biologically plausible mechanisms through which adiponectin may modify host responses, rather than as proof of direct clinical causation. Abbreviations: AdipoR1, adiponectin receptor 1; AdipoR2, adiponectin receptor 2; AMPK, AMP-activated protein kinase; NF-κB, nuclear factor kappa B; JNK, c-Jun N-terminal kinase; eNOS, endothelial nitric oxide synthase; RANKL, receptor activator of nuclear factor kappa B ligand; OPG, osteoprotegerin; PPARα, peroxisome proliferator-activated receptor alpha.

Overall, the interplay between adiponectin isoforms, receptor distribution and signalling mechanisms provides a biologically plausible framework for understanding the influence of adiponectin on local inflammation, vascularity and bony parameters within the periodontal tissues. However, it does not inherently confirm a uniform protective effect or validate a clinical utility of this system within periodontitis. The broad organization of adiponectin isoforms, receptor distribution, and major downstream pathways relevant to periodontal inflammation and bone remodelling is summarized in Figure 1.

### 2.3. Systemic Roles Relevant for Periodontitis

The systemic view considers adiponectin as an insulin-sensitizing, vasculo-protective and immune-modulating adipokine which seems to offset several detrimental characteristics of obesity and insulin resistance [1,2,5,6,7]. Low serum adiponectin is frequently accompanied by obesity (particularly central abdominal fat distribution), insulin resistance, impaired glucose tolerance, dyslipidaemia, endothelial dysfunction and chronic low-grade inflammation [3,7,15,16]. These links established adiponectin relevance to the wider multimorbidity context which currently frames the debate about periodontal disease.

From the inflammatory perspective, adiponectin might exert regulatory control on systemic inflammation via its effects on cytokine production, macrophage polarization and clearance of apoptotic material and a wider array of resolution-associated pathways [1,7,9]. A decrease in adiponectin would tilt the balance toward a more ‘permissive’ inflammatory state. This might represent a background in obesity and type 2 diabetes mellitus that predisposes the peripheral tissues, including the periodontium, to an exaggerated inflammatory response. However, this does not imply that adiponectin ‘causes’ periodontitis, but rather that adiponectin-dysregulation could be part of a systemic milieu where periodontal destruction becomes more probable or more severe.

Adiponectin has also been linked to vascular and skeletal pathway that could be pertinent to periodontitis. Data from experimental models showed that adiponectin can enhance endothelial function, increase nitric oxide bioavailability and down-regulate expression of adhesion molecules under specific conditions [1,2,7]. It has been correlated with regulation of osteoblast differentiation, osteoclastogenesis and with modulation of the RANKL/osteoprotegerin balance [7,11,12,17,18,19]. Such biological rationale can easily be linked to periodontitis inflammation and alveolar bone loss, although establishing the net effect in human periodontitis may still be a context-dependent factor.

Overall, adiponectin should be considered a biologically relevant mediator in a systemic immuno-metabolic environment and as potentially having a role in mediating host response in periodontitis, especially in metabolic dysfunction state, without overlooking that adiponectin might be just modifying the clinical features of periodontitis that is essentially initiated by the microbial agent.

## 3. Periodontitis Within the Adipokine-Rich Systemic Milieu

### 3.1. Brief Overview of Periodontitis Pathogenesis

Periodontitis is initiated by dysbiotic dental biofilms in the gingival sulcus and periodontal pocket, but destruction of periodontal tissue occurs mainly via the host inflammatory response to this challenge rather than the effect of bacteria per se [1,2]. This microbial challenge, in susceptible individuals, causes continued activation of neutrophils, macrophages, lymphocytes, and resident stromal cells, leading to secretion of pro-inflammatory cytokines, chemokines, matrix metalloproteinases, prostanoids, and other mediators that cause connective tissue degradation and alveolar bone resorption [1,2,6,16]. Hence the critical etiologic point is that periodontitis is initiated by a biofilm. The extent of tissue destruction however is critically modified by the host inflammatory status and host general factors. The advent of osteoimmunology has deepened the knowledge by stressing how immune signals and bone turnover interaction occurs at the periodontal lesions; the signalling is based on the principle that pro-inflammatory mediators and receptor activator of nuclear factor kappa B ligand (RANKL) support differentiation and activation of osteoclasts while regulatory mechanisms favour resolution and stability. During periodontal lesions the signalling shifts toward chronic inflammation and uncoupled bone resorption. This context is relevant in the study of adiponectin in that whatever can influence the inflammatory tone, the vascular environment or osteoclast signals can potentially modify the disease without having an etiologic role. The consequences of periodontitis on the general organism have also been observed, in fact bacterial products and inflammatory signals can enter the systemic circulation and contribute to the low-grade systemic inflammation, whereas the systemic metabolic or inflammatory conditions may influence the periodontal lesion [2,5,6]. This bi-directional signalling constitutes the scientific rationale for the study of adipokines in periodontal lesions, although adipokines cannot substitute for bacterial dysbiosis in its etiologic role.

### 3.2. Obesity, Type 2 Diabetes Mellitus, and Adipokine Imbalance in Periodontitis

Increased prevalence, severity or therapeutic resistance of periodontitis has been consistently found to correlate with obesity and type 2 diabetes mellitus, and to remain so when adjusted for at least some behavioural risk factors they share [2,5,6]. A suggested biological mediator of this co-morbidity between periodontitis, obesity and diabetes is modified adipokine signalling. Obesity (especially visceral) is now thought to represent a hypertrophic, hypoxemic and macrophage-infiltrated state of adipose tissue with altered secretion of adipokines and cytokines, notably with decreased adiponectin and increased leptin or resistin [2,5,6,16]. Insulin resistance, impaired glycemic regulation, increased oxidative stress and a generally low-grade chronic inflammation in type 2 diabetes mellitus likely exacerbate this altered systemic environment further.

Most human studies of periodontitis fall within this general theme: lower adiponectin and/or higher leptin, or a leptin/adiponectin ratio favouring increased leptin relative to adiponectin, has been more common in subjects having both periodontitis and obesity or diabetes than in periodontitis alone [3,4,5,15,20,21,22,23,24,25,26,27,28,29,30,31,32]. These results tend to support the idea that periodontal inflammation arises, for the most part, within a background of impaired immunity-metabolism; they are not, however, evidence that modified adipokine status causes periodontitis, and can instead be thought to support a role of altered adipokines as one factor contributing to systemic milieu-mediated increased vulnerability to or poor resolution of periodontitis.

The modified adipokine status is dynamic, with improved metabolic control and periodontal therapy associated in some cases with adiponectin-preferring patterns following weight loss and periodontal treatment [15,26,31,33,34,35,36,37], but not universally, and with an unavoidable confounding that multiple factors change. These further reinforce cautious interpretation of the results: adiponectin might be considered as a variable within systemic host modulation which is context-dependent, rather than being indicative of a unique disease-specific feature.

### 3.3. Conceptual Role of Adiponectin in the Perio-Systemic Axis

Within this concept, adiponectin can be thought of as a potential modulator of the perio-systemic axis rather than a key regulator or primary effector of a disease state. In numerous research settings (experimental and clinical), it is associated with less inflammatory signalling, increased endothelial function, and a less osteoclastogenic microenvironment [1,2,5,6,7,9]. During a metabolically unfavourable state (e.g., abdominal obesity, type 2 diabetes) the lack of adiponectin signalling may remove some inhibition and result in a more osteoclastogenic environment that promotes periodontitis.

However, this is clearly a limited paradigm. The roles of systemically produced vs locally derived adiponectin have not been clarified, receptors might behave differently in various tissues, and some mechanistically driven studies do not concur with an anti-inflammatory effect. Therefore, adiponectin should not be seen as universally beneficial. A more appropriate conclusion would be that adiponectin is a biologically significant yet context-dependent mediator whose importance varies across various compartments, disease states, and study types.

## 4. Human Evidence on Adiponectin in Periodontal Health and Disease

### 4.1. Circulating Adiponectin in Periodontitis

There seems to be a directional trend to adiponectin levels in periodontitis, but no uniform signal, in human studies assessing circulating adiponectin. Across studies (case–control, cross-sectional, selected interventional) it is usually the case that circulating adiponectin levels are lower in periodontal disease than in periodontal health, while circulating leptin and/or the leptin/adiponectin ratio is higher (especially in cases of concomitant type 2 diabetes mellitus and/or obesity) [3,4,5,15,20,21,22,23,24,29,30,33,34,35,36]. This pattern is biologically reasonable within a broader immuno-metabolic context, but it is too simplistic to just focus on this aspect of things. The degree of association varied hugely between cohorts and was dependent on factors such as how periodontitis was defined, the body composition, glycemic state and smoking history of participants, whether the participant was taking medication and how circulating adiponectin (or related indices) were measured. Meta-analyses confirmed the link between low adiponectin and periodontal disease but highlight considerable heterogeneity between studies [3,4,5]. Therefore, it is perhaps most appropriate to think of low circulating adiponectin as an index of the broader context in which the participant is prone to developing periodontal disease rather than a feature that characterizes all cases. Adiponectin is unlikely to be specifically a periodontal marker itself. The relationship seems stronger where type 2 diabetes mellitus and/or obesity are present, and studies looking at groups with and without these metabolic conditions consistently find the lowest adiponectin levels in those with the double condition (e.g., obesity + periodontal disease) [15,21,23,24,29,30,34,35,36]. This observation seems consistent with the view that there is feedback between periodontal disease and metabolic dysfunction via shared hormonal and inflammatory mechanisms. However these are predominantly observational studies, and it is impossible from these alone to say whether low adiponectin plays a causal role in periodontal breakdown or is simply a marker of the metabolic environment, or both. Looking at factors other than total adiponectin may provide more information, for example, adiponectin quality. High molecular weight adiponectin/total adiponectin has been reported to be positively associated with periodontal disease in some cohorts [8]. However, these studies are very few and clearly highlight that there is nothing quite so simple as just comparing adiponectin levels and the clinical parameters associated with periodontitis. Changes in circulating adiponectin have been reported following non-surgical periodontal treatment, typically small increases, especially in the case of type 2 diabetes mellitus and/or obesity [15,33,34,35,36,38]. These studies are interesting as they suggest that the decrease in inflammation following periodontal treatment may lead to an increase in adiponectin, but the changes are not large or ubiquitous, and other co-variables that may also affect adiponectin are unlikely to have been fully controlled for. Thus, for now circulating adiponectin appears to be best understood as a context-specific systemic biomarker within the host–metabolic interaction.

### 4.2. Local Adiponectin in Saliva, Gingival Crevicular Fluid and Gingival Tissue

Local oral measurements provide another, though not necessarily more accurate, picture. Adiponectin has been measured in saliva, gingival crevicular fluid (GCF), and gingival tissue and in many cases local adiponectin is reported as decreased in periodontitis compared to periodontal health [10,25,26,27,28,30,31,37,39,40]. Local readings are potentially more relevant in so far as they may better reflect alterations in the local inflammatory environment compared to circulatory markers. Local readings themselves however are subject to methodological constraints and cannot be readily equated with circulatory measures.

With respect to local oral readings generally, salivary samples in particular often show a decrease in adiponectin accompanied by increases in other pro-inflammatory cytokines such as leptin, resistin or calprotectin in periodontitis, with the observation that the combination of analytes may better differentiate the clinical state than individually measured parameters [25,26,28,32,37] and suggesting that adiponectin may best be used as part of a panel of oral markers. The interpretation of salivary adiponectin is complicated by a complex and variable medium (saliva) the characteristics of which (e.g., flow, method of collection, dilution, level of oral hygiene, contributions from salivary glands, body state, etc.) can vary significantly between individuals and situations.

The GCF offers a potentially more tissue-specific milieu than saliva and it has been reported that adiponectin levels in the GCF are decreased, and pro-inflammatory markers increased at sites affected by periodontitis. GCF levels for both may return toward baseline values following periodontal therapy [27,28,30,31]. These observations are certainly in keeping with altered local adipokine balance in chronic periodontal inflammatory disease, but GCF studies are limited by their typically small sample sizes and inconsistencies in the methods of sample collection (sites measured, time intervals), analysis and the reporting of data [40].

The local measurement of adiponectin expression in periodontal tissues may be mechanically important as both adiponectin and its receptors have been shown to be expressed by local tissues in periodontitis [10,40]. Such findings, however, do not shed light on the interplay between circulating and locally produced adiponectin in the context of the periodontal environment.

The picture of altered adiponectin function in periodontitis provided by local readings may therefore not necessarily apply to all adiponectin and the assumption that measurements made at the different local compartments, i.e., salivary, GCF and tissue, are equivalent should be made with great caution.

### 4.3. Major Modifiers and Sources of Heterogeneity in Human Studies

Interpretation of human adiponectin measurements for periodontitis is confounded by numerous systemic and methodological modifiers of adiponectin biology. Obesity is arguably the strongest and most dominant. Strongly correlating with lowered adiponectin and dysregulated adipokines, obese status can have a large impact on both circulating and localized measures [2,3,4,5,6,21,24,29,31]. Type 2 diabetes mellitus is another very strong modifier as glycemic control, duration, medication use and insulin resistance can all affect circulating adiponectin independently of the periodontal status [15,23,30,34,35,36].

Other significant modifiers include cigarette smoking, age, sex, inflammatory and nutritional status, as well as therapy with statins or antidiabetics [3,4,5,6,8,32,41,42]. Such factors are not uniformly controlled among studies; the obese state or diabetes might potentiate the periodontal effect in one study group but obscure it in another. This alone might explain the lack of concordant conclusions in all data sets.

Methodological variance adds another layer of variability. Methodologies differ with regard to case definition, staging, source sample, assay platform and unit of measure as well as what measurement is analyzed (e.g., total, HMW, or a ratio-based adiponectin measure) [3,4,5,8,25,26,27,28,39]. These differences are not trivial as they may relate to different compartments of a pathway (e.g., total, HMW, Salivary and GCF adiponectin might reflect different processes and should not be used as interchangeable readouts of one signalling pathway).

As such the human evidence is more indicative of a role for dysregulated adiponectin within a negative immunometabolic milieu than evidence for a direct or periodontal-specific causative role.

### 4.4. Genetic and Epigenetic Aspects

Genetic variance in adiponectin-related pathways has started to be investigated with respect to periodontitis. A case–control study by Borilova Linhartova et al. tested the association of polymorphisms in several adipokine genes, including *ADIPOQ*, with chronic periodontitis and circulating adipokine levels [41]. Several of the alleles tested were associated with a changed adiponectin and leptin profile, but only weakly with status of periodontal disease, indicating that inherited variance contributes to periodontitis risk on an inflammation–metabolic context, rather than a definitive disease-specific marker. Gene-environment interactions have been investigated in greater detail by Cao et al., who looked at the interaction of either moderate or severe periodontitis with specific *ADIPOQ* (rs1501299) and *LEPR* (rs1137100) polymorphism and type 2 diabetes mellitus risk [42]. Their results lend support to the notion that adipokine gene variation interacts with periodontitis inflammation on a general metabolic disease context; nevertheless, these results are limited and there is still a lack of clarity as to how to apply genetic variation to periodontal risk or mechanical understanding. Little direct epigenetic evidence regarding adiponectin signalling within periodontal tissue exists. While hypomethylation and microRNA-mediated regulation of adiponectin signalling have been established in several other inflammation–metabolic diseases [7,9,43], analogous evidence is lacking in the context of the periodontium. At present, genetic and epigenetic findings are best interpreted as speculative additions to the adiponectin–periodontitis discourse, rather than clinical interpretations.

### 4.5. Human Evidence: What Can and Cannot Be Concluded at Present

A careful appraisal of the human data, however, allows for four principal conclusions to be made: First, disordered adiponectin profile is frequently seen in periodontitis in the presence or absence of comorbid type 2 diabetes mellitus or obesity. Second, this profile exists in both circulation and local compartment and, although a positive association exists, they cannot be regarded as interchangeable surrogates of one another. Third, the data remains highly variable since adiponectin levels are affected by strong confounders and by large variations in methods. Fourth, human data does not demonstrate adiponectin to be a disease-specific biomarker, a causative factor, or a therapeutically responsive target [3,4,5,15,20,21,22,23,24,25,26,27,28,29,30,31,32,33,34,35,36,37,38,44].

In this light, the primary importance of the human data is to establish adiponectin as one player within the network of the immunometabolic link between periodontitis and metabolic comorbidity, a finding of scientific interest rather than clinical utility at the present. Key characteristics of the human evidence are summarized in Table 1, with emphasis on study design, modifiers, bio-logical compartment, adiponectin-related measure, and the main interpretive limitations relevant to clinical translation.

## 5. Mechanistic Insights from Experimental Models

Experimental models have generated mechanistic and proof-of-principle data that can illuminate aspects of the human literature but do not answer key questions about causality, compartment specificity or clinical applicability. Instead, the models may serve to clarify how adiponectin signalling can affect inflammatory, stromal, immune, vascular and bone relevant pathways under certain conditions and should be considered primarily in terms of biological plausibility.

### 5.1. Adiponectin Receptors in the Periodontal Tissues

Adiponectin receptors are expressed in oral tissues and periodontal tissues thereby establishing structural integrity of local signal transduction pathway responsiveness to adiponectin. Studies have identified adiponectin receptors AdipoR1 and AdipoR2 in gingival tissues; more specifically in the epithelial layer and connective tissue components of the gingival tissues as well as in cultured periodontal ligament fibroblasts and gingival fibroblasts [10,11,12,13,45,46]. These finding suggest the periodontal tissues themselves have a capacity to respond to adiponectin signalling not simply by passive exposure to circulatory adipokines.

Furthermore, receptor expression does not imply an identical cellular response. Different receptor expression levels might be modulated by various inflammatory stimuli, the individual’s metabolic status and cell types, while the signalling outcomes may differ for various cell types when their respective receptors are activated. Thus, these receptor expression studies do indicate biological plausibility without necessarily demonstrating whether signalling is either protective, compensatory, maladaptive or context-specific within the periodontal tissues.

### 5.2. In Vitro Effects on Resident Periodontal Cells

In vitro experiments using gingival fibroblasts and periodontal ligament cells indicate that adiponectin has the potential to modulate several pathways of periodontal tissue homeostasis such as cytokine signalling, extracellular matrix modulation, wound-healing responses, osteogenic differentiation and RANKL/osteoprotegerin balance [11,12,13,45,46]. In some experimental settings, adiponectin reduces inflammatory mediator expression or suppresses lipopolysaccharide-induced signalling, findings that are compatible with an anti-inflammatory or pro-resolving role. Other studies indicated positive effects on regenerative or osteogenic properties under specific conditions, again supporting potential modulation by adiponectin of host repair pathways [11,12,13,45].

However, the adiponectin effect in periodontal cells is not consistent throughout the literature. In at least one report, adiponectin was shown to increase the expression and secretion of IL-1, IL-6, IL-8 without parallel induction of anti-inflammatory cytokines like IL-10 [46]. This is an important issue, as it challenges any possible claim of a uniform anti-inflammatory effect of adiponectin on periodontal tissues. Based on such conflicting results, the most reasonable conclusion would be not that adiponectin has good or bad effects in periodontal tissues, but that these effects are variable and depend on the experimental model, cell type, adiponectin form, dose, timing, receptor environment, and stimulus.

These are important limits, as cell culture studies do not truly represent the niche, which is populated by polymicrobial biofilms, host cells, blood vessels, the extracellular matrix and the complexity of the inflammatory response in vivo. The doses used and isoforms available are not always representative of the quantities and types of adiponectin in the human tissues; thus, their interpretation for clinical periodontitis patients cannot be taken as directly applicable evidence.

### 5.3. In Vitro Effects on Immune and Vascular Cells

Direct periodontal evidence, including those studies of classical immune and vascular cell populations, has been less extensive than studies utilizing resident stromal cell populations. However, by extrapolating from general adiponectin biology, one can hypothesize several relevant functional pathways. Adiponectin has been reported to influence altered macrophage polarization, suppression of certain inflammatory mediator production, enhanced endothelial cell function and nitric oxide availability, and alteration of ceramide signalling pathways in non-periodontal systems [1,2,7,9,14,43]. By extension, these can be expected to influence leukocyte recruitment and/or adhesion within periodontal lesions, inflammatory feedback amplification, microvascular dysfunction, and alveolar bone osteoimmune phenomena.

Some periodontological studies appear consistent with these concepts. Applications of adiponectin receptor agonists to diabetic periodontal models resulted in altered macrophage infiltration, chemokine production, and osteoclastogenic pathway mediation, attenuated osteoclast formation and subsequent alveolar bone resorption [18,19,47]. This suggests a degree of plausibility of adiponectin signalling affecting immune–stromal interactions and bone-related inflammatory mediators in poor metabolic environments.

However, direct periodontal evidence is limited, and extrapolations of the relevant cellular and molecular mechanisms were often made from related literature. Therefore, the proposed physiological effects on the periodontal lesions should be considered hypothesis-generating.

### 5.4. Animal Models of Periodontitis and Metabolic Disease

The strongest preclinical data suggesting that manipulating adiponectin signalling experimentally can affect periodontal outcome in a controlled manner come from animal models. Administration of adiponectin or adiponectin receptor agonists has been shown to be associated with less inflammatory infiltrate, less osteoclast activity, favourable balance between RANKL and osteoprotegerin, and reduced alveolar bone loss in obese or diabetic models of ligature-induced periodontitis [17,18,19,47]. These results are consistent with the hypothesis that pathways involved in adiponectin signalling may regulate periodontitis.

However, caution must be applied when interpreting these animal studies. These are generally rodent, acute studies in animal models of metabolic disease and periodontal injury, neither of which truly mimic the chronicity, behavioural intricacies, pharmacotherapy burden, nor the microbiota involved in human periodontitis. Furthermore, the use of systemic agonists in animal studies affects a wide array of tissues, not necessarily limiting effect to the oral environment, and it is difficult to ascertain how much of any observed effect is local versus how much it reflects broader metabolic or inflammatory effects.

Therefore, while animal models provide proof of principle and justify ongoing interest in mechanisms, they cannot be interpreted as establishing a clinical efficacy, safety, or human readiness profile.

Three conclusions can be drawn cautiously from the experimental literature. First, adiponectin signalling is biologically relevant to oral/periodontal tissues given the expression of receptors locally, and which can be modulated by inflammatory stimuli [10,11,12,13,40,45,46]. Second, adiponectin is biologically active to influence pathways involved in the production of inflammatory mediators, matrix modulation, osteoimmune balance and alveolar bone loss [11,12,13,14,17,18,19,45,46,47]. Third, adiponectin signalling seems context-dependent and not ubiquitously beneficial with effects varied depending on the model and experimental situation.

### 5.5. Mechanistic Evidence: What Can and Cannot Be Concluded

**Figure 2:**
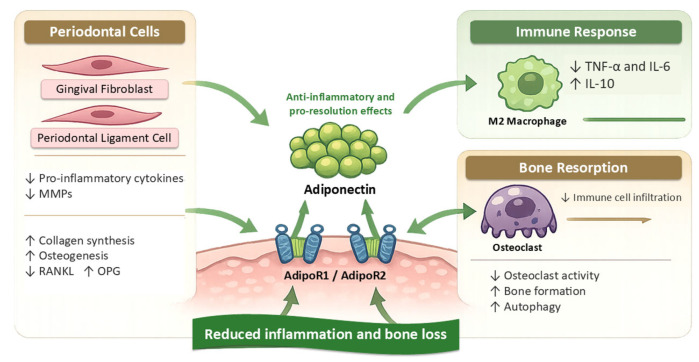
Experimental framework showing how adiponectin-related signalling may influence oral and periodontal cell responses, inflammatory pathways, and alveolar bone loss in mechanistic models. This figure integrates the principal mechanistic observations derived from in vitro and animal studies. Adiponectin and adiponectin receptor agonists have been reported, under selected experimental conditions, to influence resident oral and periodontal stromal cells, inflammatory mediator production, macrophage-related signalling, osteoclastogenesis, and alveolar bone loss. The figure is intended as a schematic summary of experimental and preclinical findings only. It does not imply that adiponectin is a primary cause of periodontitis, nor that these mechanisms have been validated as clinically actionable therapeutic pathways in humans. Where arrows indicate suppression of osteoclastogenesis or reduced alveolar bone loss, these reflect observations from specific mechanistic or animal models and should be interpreted as proof-of-principle rather than clinical evidence. Abbreviations: PDL, periodontal ligament; AdipoR, adiponectin receptor; IL, interleukin; TNF-α, tumour necrosis factor alpha; NF-κB, nuclear factor kappa B; RANKL, receptor activator of nuclear factor kappa B ligand; OPG, osteoprotegerin.

What cannot yet be concluded is equally important. Experimental data do not establish that adiponectin is a causal driver of periodontitis in humans, that adiponectin supplementation would be clinically beneficial, or that receptor agonism is close to therapeutic application. The principal contribution of this literature at present is therefore mechanistic clarification and translational hypothesis generation. Figure 2 schematically integrates the principal mechanistic observations derived from in vitro and animal studies and provides the experimental basis for the cautious translational discussion that follows.

## 6. Therapeutic and Translational Perspectives

Section 5 should be interpreted against the background that adiponectin-related findings in periodontitis range from observational human associations to mechanistic and preclinical proof-of-principle studies. These evidence layers are not equivalent. The current literature supports biological and translational interest, but not clinical readiness.

### 6.1. Periodontal Treatment and Adiponectin Levels

A number of publications have implied the potential for some small changes in adiponectin profile to occur with non-surgical periodontal treatment, particularly in the metabolically compromised patient. In some study groups, periodontal therapy was associated with an increased circulating adiponectin, a healthier leptin/adiponectin ratio, or advantageous changes in adipokines locally. The potential interest is because this is compatible with the concept of adiponectin responding to a decreased inflammatory burden [15,33,34,35,36,37,38].

However, this should not be considered an overstatement. Changes are almost uniformly of small magnitude, are not present in all studies and, given their potential association with improvements in glucose metabolism, weight management, improved compliance, or other lifestyle changes, are not readily attributable solely to therapy. Conventional mechanical periodontal treatment supplemented with risk factor control is current standard non-surgical periodontal management; the evidence does not support the routinely monitoring of adiponectin in the management of clinical periodontal disease or that it constitutes a treatment target in routine treatment.

### 6.2. Adiponectin Receptor Agonists and Host Modulation

The mechanistically informative, preclinical testing of exogenous adiponectin or adiponectin receptor agonists including AdipoRon and AdipoAI, justifies ongoing scientific interest in the molecule and its receptor. In obese and/or diabetic animal models of periodontitis, such treatments were shown to attenuate inflammatory infiltration, decrease osteoclast activity, and decrease alveolar bone loss. Collectively, these studies provide biological feasibility for a mechanism by which adiponectin signalling affects inflammatory periodontal pathways in metabolically maladaptive states [17,18,19,47].

These data remain preclinical, however. They were generated under controlled, laboratory settings with highly limited comorbidities, relatively brief timeframes, and dosing regimens that cannot be extrapolated to human medicine. No clinical trials have assessed the efficacy of adiponectin receptor agonists in periodontitis. As such, these molecules remain as investigational tools for mechanistic inquiry and to assess host-modulatory approaches, rather than a near-term clinical prospect.

### 6.3. What Would Be Required for Meaningful Translation?

Translational application of adiponectin-based therapies raises several concerns which would have to be overcome before it could be of clinical use. These include: First of all, a definition of relevant targets would have to be more clearly established. The clinical use of an adiponectin-based therapy may only be feasible in metabolically compromised subpopulations of patients rather than an unselected periodontal patient group (e.g., obese patients, patients with type 2 diabetes mellitus, or systemic inflammatory diseases).

Secondly, the mode of administration would have to be established. Systemic administration would necessarily alter metabolism, vascular biology, immune responses, and the skeleton as well as affect other, seemingly unrelated pathways. This is likely to lead to off-target effects and a necessity for extensive dosage trials as well as long-term safety analysis. A localized periodontal application of an adiponectin-based therapy could potentially limit the effects of an adiponectin-based treatment to the periodontium and the surrounding tissue; however, this has, as of yet, not been investigated and would necessitate studies on pharmacokinetics, toxicology and formulation studies before human application.

Thirdly, the evidence pathway required before any treatment that can be of clinical use would have to be extremely extensive; only after confirmation of underlying mechanisms by studies in experimental models and improved standardization of human biomarkers followed by phase I studies investigating safety, feasibility, biological responses and suitable endpoints could application in a relevant clinical situation be envisioned. This is still a long way away.

### 6.4. Biomarker Relevance in Personalized Periodontal Care

Adiponectin remains interesting, as a component of overall panel assays, not a discrete clinical marker. Several studies have indicated a changed pattern of adiponectin associated with periodontitis in humans, particularly when accompanied by type 2 diabetes mellitus or obesity, with combined adipokine patterns proving more informative than any single parameter. Unfortunately, there is considerable overlap between the state of health and disease, and levels can be profoundly altered by systemic and methodological modifiers [3,4,5,8,25,26,27,28,29,30,31,32,33,34,35,36,37,38,39,44].

Therefore, it is far too early to consider adiponectin a practically relevant biomarker in routine diagnosis, prognosis or monitoring of treatment. It is more likely that any translation role to the clinic will be through composite biomarkers that include a mix of adipokines, metabolic markers, inflammation and specific periodontal measurements in the future. The future possibility itself remains contingent upon improved standardization, prospective verification and defined compartment-specific use.

Overall, the translational message of the literature is best summarized as follows—adiponectin is biologically relevant and scientifically interesting, but adiponectin-based biomarker use and adiponectin-targeted therapy remain investigational. To distinguish more clearly between translationally relevant human evidence and preclinical mechanistic findings, representative studies are summarized separately in Table 2A,B.

## 7. Methodological Limitations and Knowledge Gaps

### 7.1. Limitations of Human Evidence

Majority of human investigations are cross-sectional or case–control, performed in mostly small single-centre cohorts [3,4,5,8,15,20,21,22,23,24,25,26,27,28,29,30,31,32,33,34,35,36,37,38,44]. While it is possible for these study designs to find association between two variables, they are unable to ascertain any sense of temporality or causation. As such, it is impossible to deduce whether modifications in adiponectin precede the development of periodontal destruction, are a direct result of increased inflammatory burden, a reflection of concurrent metabolic dysregulation, or an amalgamation of these possibilities.

In addition, inconsistencies in periodontal case definition and phenotype abound, using either former classifications or present staging and grading protocols, and the reporting of clinical measures such as probing depth, clinical attachment loss, bleeding, radiographic bone loss and disease extent is far from uniform, which makes interpretation and cross-cohort comparisons difficult and may partly explain some discrepancies in the association of adiponectin and periodontitis.

Systemic confounders provide a significant hurdle. Obesity, distribution of fat depots, insulin resistance, duration of diabetes, level of glycemic control, use of tobacco products, age, gender, nutritional status, and certain medications, background inflammation, etc., influence the biology of adiponectin [2,3,4,5,6,8,32,41,42]. Characterization of these variables has not been well documented in most investigations. Hence, the extent to which modifications of adiponectin can be regarded as periodontitis-specific and not just a surrogate for the existing metabolic background remains unclear.

Measurement heterogeneity is another drawback. Different sources for samples (plasma, serum, GCF, saliva), measurement time-point, collection protocol, assay platform used, unit of measurement, what variable is being reported (total adiponectin, HMW adiponectin, ratio of some adiponectin subtypes, salivary expression, tissue expression) varies from one study to the next [3,4,5,8,25,26,27,28,39] and these parameters are certainly not interchangeable as a concept, under a general label of adiponectin.

### 7.2. Limits of Mechanistic and Preclinical Models

Experimental studies can help elucidate mechanisms but simplify the complex biology of human periodontitis. Models using cultured cells do not replicate the full tissue architecture and host/microbe complexity found in periodontal lesions, and animal models, where used, often compress development to limited time courses and simplified metabolic environments [11,12,13,17,18,19,45,46,47]. The dose, concentration, and duration of adiponectin exposure in experimental models may differ significantly from those encountered in human physiology. Sex-specific differences, chronic medication burden, background comorbidity and long-term safety concerns are seldom modelled. All of these issues are important since endocrine, metabolic and inflammatory environments profoundly impact the biology of adiponectin and the therapeutic potential of it and its analogues. Therefore, a significant translation gap between experimental proof-of-principle studies and human clinical utility remains and experimental models remain primarily for mechanistic and proof-of-concept systems, not necessarily for prediction of clinical dose, safety, or impact size in humans.

### 7.3. Outstanding Conceptual Questions

There are many conceptual questions still to be addressed. Most notable are issues of compartment specificity. The precise relationship between circulating, salivary and GCF adiponectin, expression at the tissue level and receptor activity in vivo, and which compartment is most biologically relevant to periodontitis is not yet defined. In addition, the relevant role of total and HMW adiponectin, and ratio measures is not clear.

The issue of cause and effect versus association is also currently under investigation. Current data clearly points to an association between abnormal adiponectin function and periodontitis, in the general context of immunometabolism, and particularly obesity and type 2 diabetes, but does not prove whether adiponectin is a causal factor, a compensatory mechanism, an indicator of other metabolic disturbances, or a mixture of the above.

Thirdly, the direction of action of adiponectin in the periodontium is still debated. Most evidence is compatible with an anti-inflammatory or regulatory role of adiponectin; however, a few observations appear paradoxical or even pro-inflammatory under specific circumstances [46]. This would mean that the biological relevance of adiponectin in periodontitis can no longer be simplified to one single protective mechanism.

These methodological and conceptual drawbacks prevent the use of adiponectin, at present, as a biomarker for diagnosing, prognosticating or treating periodontitis, and clearly distinguish between hypotheses that can be backed up by the relevant mechanistic model and conclusions that are clinically substantiated.

However, they do not nullify the importance of research in the field and rather define the scope of future work. It is already proven that adiponectin is relevant enough to justify continued research and analysis, although the evidence so far is insufficient for its use in clinical practice.

### 7.4. Research Priorities Moving Forward

Prospective, better standardized and better phenotyped human studies should be the focus of future work, using contemporary definitions of periodontitis and more precise characteristics of obesity and metabolic state, along with improved controls for confounds (e.g., smoking, drugs, diet) where possible. Direct compartment comparisons of readouts would be valuable for distinguishing between serum-, saliva-, GCF- and tissue-level adiponectin information.

Prospective studies, including interventional trials, are needed to assess if any specific adiponectin readouts change appropriately with progression of periodontal disease or treatment outcomes, or with metabolic changes over time. Mechanistic work should also increasingly rely on combined systems approaches such as co-culture or organotypic models more relevant to the periodontal niche, whereas translational studies should first consider issues of safety, delivery mechanism, subject population and compartment-specificity.

The main unknown is not the relevance of adiponectin in biology, but rather what that relevance means for various compartments, diseases and levels of evidence.

## 8. Future Research Agenda

Future studies of adiponectin in periodontitis should strive to transition from studies with broad correlative data toward better standardized human studies, more precise, compartmental-specific interpretation, and better correlation between mechanistic and clinical data. Critical is the establishment of large, well-characterized prospective cohorts based on contemporary periodontal definitions and clear specification of obesity, insulin resistance, glycemic status, smoking, medications, and nutritional condition [2,3,4,5,8,20,21,22,23,24,29,33,34,35].

Longitudinal studies, as well as interventions, will also be critical to establish if systemic or local concentrations of adiponectin correlate more consistently with clinical status over time and therapy response [3,4,5,6,8,15,29,31,33,34,35,36,38]. When developing these, it will be critical not to treat all adiponectin measures as equivalents; distinct measurements, including total adiponectin, HMW adiponectin, ratios, and site-specific, tissue or fluid measurements must be considered. Standardization of local measurements is a further priority.

Clear protocols for saliva and gingival crevicular fluid collection, normalization and reporting as well as use of uniform assays for measurement should improve between-study comparisons [25,26,27,28,30,31,37]. Comparing serum-, saliva-, GCF-, and tissue-specific measurements within individual study sites would allow a better distinction of the systemic vs. local adiponectin biology.

Mechanisms in periodontitis must evolve beyond simplistic cellular models toward more complex organotypic models including mesenchymal, epithelial, immune, and endothelial cell populations [7,9,11,12,13,43,45,46]. Likewise, studies using animals should examine more realistic comorbidity profiles over extended periods and explicitly evaluate sex-specific and systemic effects [14,17,18,19,47].

Ultimately, the clinical application of adiponectin-related therapies should await definition of clear biological targets, standardization of key measures, and rigorous identification of patient subpopulations wherein these pathways might play a role.

## 9. Conclusions

Collectively, the literature supports adiponectin as a biologically important, but context-specific, part of the immunometabolic connections between periodontitis and obesity, T2DM, and broader cardiometabolic risks. Human evidence has generally indicated low circulation or tissue levels of adiponectin in periodontitis (especially among the metabolically compromised); and experimental evidence has presented plausible mechanisms for how adiponectin signalling could influence inflammation, matrix integrity, osteoclast formation, and alveolar bone resorption. The literature, however, has often been inconsistent between different biological compartments and research designs and has not clearly identified adiponectin as a cause of periodontitis.

This body of literature therefore strongly favours an association, biological relevance and mechanistic interest than direct causality or clinical usefulness. Current standard periodontal care remains on traditional diagnosis, mechanical disruption of bacterial biofilm and targeting recognized lifestyle and systemic risk factors. Adiponectin should not be utilized as an isolated biomarker, routine measure, or established treatment target in periodontitis.

The current value of adiponectin research may primarily be to advance our understanding of the interaction between inflammation in periodontitis and system metabolic dysfunction, especially in the presence of obesity and T2DM. More standardized human evidence, clear compartmental differentiation of local and systemic adiponectin, adequate control of confounds, and appropriate differentiation of experimental, preclinical and clinically verified findings are necessary to advance this field. In this context, adiponectin might best be considered as an interesting potential mediator along the broad perio-systemic axis rather than as a specific treatment for periodontitis.

## Figures and Tables

**Table 1:** Key human studies evaluating adiponectin-related measures in periodontitis, including study design, modifiers, biological compartment, and principal interpretive finding.

First Author, Year [Ref]	Study Design/Population	Major Modifiers	Biological Compartment	Main Finding/Interpretive Note
Preethanath, 2024 [20]	Case–control; adults with chronic periodontitis vs periodontally healthy controls	Systemically healthy	Serum	Lower serum adiponectin and higher leptin-to-adiponectin ratio were reported in periodontitis. Supports association, but not periodontal specificity.
Mendoza-Azpur, 2015 [21]	Cross sectional; normal weight and obese adults with and without chronic periodontitis	Obesity	Serum	Obesity-associated periodontitis showed the most unfavourable adipokine profile, consistent with strong metabolic modification of periodontal findings.
Thanakun, 2016 [22]	Cross sectional; Thai adults with and without periodontitis	Overweight and obesity common	Serum	Periodontitis was associated with reduced adiponectin and increased C reactive protein after adjustment for age, sex and body mass index.
Jing Ling, 2014 [23]	Case–control; type 2 diabetes, chronic periodontitis, both, or neither	Type 2 diabetes	Serum	The leptin to adiponectin ratio was highest in subjects with combined diabetes and periodontitis and was associated with both glycemic and periodontal severity.
Zimmermann, 2013 [24]	Four group design; obese and normal weight subjects with and without chronic periodontitis	Obesity	Serum, gingival crevicular fluid	Lowest adiponectin levels were reported in participants with both obesity and periodontitis, supporting interaction between periodontal and metabolic context.
Nagano, 2011 [8]	Cross-sectional; middle-aged men from a community cohort	General population	Serum	Suggests that adiponectin quality, not only total concentration, may be relevant to periodontal status.
Kardeşler, 2010 [35]	Interventional; patients with type 2 diabetes and chronic periodontitis receiving non-surgical periodontal therapy	Type 2 diabetes	Serum	Periodontal treatment was associated with increased adiponectin and reduced pro-inflammatory cytokines, together with improvements in periodontal indices.
Gonçalves, 2015 [33]	Interventional; patients with type 2 diabetes with and without chronic periodontitis undergoing scaling and root planning	Type 2 diabetes	Serum	Nonsurgical periodontal therapy improved clinical periodontal parameters and was accompanied by favourable changes in adipokine profile, including higher adiponectin.
Boyapati, 2018 [29]	Case–control; obese and normal weight adults with chronic periodontitis and healthy controls	Obesity	Serum	Periodontitis was associated with lower adiponectin and higher leptin, with the most pronounced imbalance in obese subjects with chronic periodontitis.
Wang, 2017 [34]	Interventional; type 2 diabetes patients with chronic periodontitis followed periodontal therapy	Type 2 diabetes	Serum	Better glycemic control and nonsurgical periodontal therapy were linked to increases in adiponectin and reductions in tumour necrosis factor alpha.
Ogawa, 2014 [15]	Interventional; individuals with type 2 diabetes and chronic periodontitis	Type 2 diabetes	Serum	Periodontal treatment improved adipokine profiles, including adiponectin, and was associated with improved glycemic control.
Sun, 2011 [36]	Interventional; patients with type 2 diabetes and chronic periodontitis receiving intensive periodontal therapy	Type 2 diabetes	Serum	Intensive periodontal therapy increased adiponectin and reduced systemic inflammatory markers and insulin resistance indices.
Mohamed, 2015 [30]	Cross sectional; adults with and without type 2 diabetes and chronic periodontitis	Type 2 diabetes	Serum	Chronic periodontitis was associated with lower adiponectin and higher leptin in diabetic subjects compared with nondiabetic controls, independent of body mass index.
Vivekannada, 2019 [31]	Interventional; overweight or obese adults with chronic periodontitis undergoing periodontal therapy with or without weight reduction	Obesity, weight loss	Gingival crevicular fluid	Combined weight reduction and periodontal therapy produced greater increases in adiponectin and greater reductions in inflammatory markers than periodontal therapy alone.
Bayirli, 2025 [25]	Cross sectional; subjects with periodontal health, gingivitis and periodontitis	General population	Gingival crevicular fluid	Salivary adiponectin decreased and leptin and calprotectin increased with disease severity; combinations of these markers improved discrimination between health and disease compared with single markers.
Sales-Peres, 2023 [26]	Longitudinal; obese individuals undergoing bariatric surgery with periodontal assessment	Obesity, bariatric surgery	Saliva	Bariatric surgery led to changes in salivary adiponectin and albumin; higher adiponectin levels were associated with better periodontal status after weight loss.
Fairlin, 2021 [27]	Case–control; chronic periodontitis patients and periodontally healthy controls before and after nonsurgical therapy	General population	Gingival crevicular fluid	Reduced local adiponectin and increased resistin were observed at periodontitis sites, with partial normalization after therapy.
Abdellatif, 2022 [37]	Interventional; chronic periodontitis patients (often with metabolic risk factors) receiving nonsurgical therapy with follow up	Obesity and or other metabolic risk common	Saliva	Periodontal therapy increased local adiponectin and reduced leptin, changes that tracked with improvements in probing depth and bleeding scores.
Borah, 2023 [28]	Cross sectional; chronic periodontitis patients vs. healthy controls	General population	Saliva	Periodontitis was associated with lower salivary adiponectin and higher resistin; adiponectin showed an inverse relationship with clinical periodontal severity.
Varma, 2024 [32]	Cross sectional; periodontitis patients with and without acute myocardial infarction and healthy controls	Cardiovascular disease	Saliva	Patients with acute myocardial infarction and periodontitis exhibited a distinct salivary adipokine profile, including altered adiponectin, compared with periodontitis alone and healthy subjects.
Yamaguchi, 2010 [10]	Case–control; gingival biopsies from chronic periodontitis patients and healthy controls	General population	Gingival tissue	AdipoR1 and AdipoR2 were expressed in gingival tissues; their expression was altered in inflamed periodontal tissues and related to clinical periodontal parameters.
Isler, 2021 [40]	Cross sectional; patients with periodontitis, peri implantitis and healthy controls	General population, implant bearing	Gingival crevicular fluid and peri implant sulcular fluid, serum	Periodontitis and peri implantitis showed distinct local and systemic adipokine patterns, with evidence of perturbed adiponectin signalling at diseased sites compared with healthy tissues.
Borilova Linhartova, 2019 [41]	Case–control; adults with chronic periodontitis and healthy controls	Genetic variation in adipokine genes	Blood, DNA	Polymorphisms in adipokine genes, including *ADIPOQ*, were associated with altered circulating adiponectin and leptin and with modest differences in periodontitis risk.
Cao, 2019 [42]	Cross sectional genetic study; Chinese adults with and without moderate or severe periodontitis and type 2 diabetes	Type 2 diabetes, *ADIPOQ* and *LEPR* polymorphisms	Blood, DNA	Individuals carrying risk genotypes in *ADIPOQ* rs1501299 or *LEPR* rs1137100 and presenting with periodontitis had an increased risk of type 2 diabetes, supporting gene environment interactions involving adipokine pathways.

**Table 2:** (**A**) Human interventional and translationally relevant studies evaluating adiponectin-related changes in periodontitis. (**B**) Experimental mechanistic and preclinical studies of adiponectin-related signalling in oral and periodontal models.

(**A**)				
**Study (First Author, Year [Ref])**	**Model/Population**	**Adiponectin-Related Intervention or Exposure**	**Periodontal Outcome**	**Key Finding/Translational Caution**
Wang, 2017 [34]	Adults with type 2 diabetes and chronic periodontitis	Conventional periodontal therapy vs no treatment, serum adiponectin measured	Periodontal therapy improved clinical periodontal status and glycemic control	Treatment increased circulating adiponectin and improved related metabolic markers, supporting biological responsiveness but not establishing adiponectin as a treatment target.
Abdellatif, 2022 [37]	Obese and normal weight adults with periodontitis	Scaling and root planning with or without adjunctive antimicrobial photodynamic therapy, whole salivary adiponectin and leptin	Both protocols improved periodontal indices, adjunctive aPDT gave additional local improvement in some parameters	Whole salivary adipokine balance shifted after therapy in obese patients, suggesting that local adipokine patterns may respond to reduced inflammation, although interpretation remains compartment-dependent.
Guo, 2025 [38]	Adults with type 2 diabetes and periodontitis, cohort followed through periodontal therapy	Nonsurgical periodontal therapy, longitudinal monitoring of TNF- α, adipokines and glycolipid markers	Periodontal therapy reduced local inflammation and improved some periodontal metrics	Therapy-associated changes in TNF-α and adipokine measures support interplay between periodontal inflammation and metabolic status, but clinical implications remain preliminary.
(**B**)				
**Study (First Author, Year [Ref])**	**Model/System**	**Adiponectin-Related Intervention or Exposure**	**Main Experimental Outcome**	**Interpretive Note/Translational Caution**
Nokhbehsaim, 2014 [12]	Human periodontal ligament cells in vitro under inflammatory and regenerative conditions	Recombinant adiponectin applied to cells, with or without pro-inflammatory stimuli	Adiponectin improved cell proliferation and wound healing related functions, and supported a regenerative phenotype	Supports regenerative and anti-inflammatory responses in vitro under selected conditions but does not provide direct clinical evidence.
Park, 2011 [45]	Human PDL and gingival fibroblasts stimulated with bacterial LPS	Globular adiponectin pretreatment before LPS challenge	Reduced LPS-induced IL 6 and IL 8 secretion in fibroblasts	Supports context-specific anti-inflammatory effects in fibroblast models but remains limited to in vitro conditions.
Wu, 2021 [13]	Human periodontal ligament cells exposed to LPS	Recombinant adiponectin added to cultures	Attenuated inflammatory response and favoured an osteogenic profile in PDL cells	Suggests dual anti-inflammatory and osteogenic effects in vitro but should not be interpreted as evidence of therapeutic readiness.
Kozak, 2025 [46]	Human PDL cells stimulated with adiponectin alone	Dose- and time-dependent adiponectin exposure	No direct periodontal outcome, in vitro cytokine profile only	Highlights that adiponectin-related effects are not uniformly protective and may vary by model conditions.
Zhang, 2014 [17]	Diet-induced obese mice and adiponectin knockout mice with ligature-induced periodontitis	Systemic adiponectin infusion in experimental periodontitis	Adiponectin reduced alveolar bone loss, osteoclast numbers and inflammatory cell infiltration in periodontal tissues	Supports proof-of-principle for adiponectin replacement in obesity-related experimental periodontitis, but not clinical translatability.
Wu, 2019 (AdipoRon) [18]	Diet-induced obese diabetic mice with experimental periodontitis	Systemic administration of AdipoRon, a small molecule adiponectin receptor agonist	Reduced alveolar bone loss and osteoclast numbers in periodontal lesions	Supports proof-of-principle for receptor agonism in preclinical diabetic models, but not therapeutic readiness.
Wu, 2022 (AdipoAI) [19]	Mouse model of type 2 diabetes-associated periodontitis	AdipoAI, a novel adiponectin receptor agonist, given systemically	Preserved alveolar bone, reduced periodontal inflammation and improved periodontal architecture	Provides preclinical mechanistic evidence linking receptor agonism with osteoclast-related pathways and bone protection.
Qiu, 2023 (AdipoAI) [47]	Diabetic rat periodontitis model with gingival fibroblast macrophage crosstalk	Systemic AdipoAI treatment, with mechanistic focus on gingival fibroblast-induced macrophage migration	Reduced periodontal inflammatory cell infiltration and mitigated bone loss in diabetic rats	Provides additional preclinical support for immune–stromal modulation in diabetic periodontal environments.

## Data Availability

No new data were created or analyzed in this study. Data sharing is not applicable to this article.

## Abbreviations

The following abbreviations are used in this manuscript:

*ADIPOQ*	adiponectin gene
AdipoAI	adiponectin receptor agonist (AdipoAI)
AdipoR	adiponectin receptor(s)
AdipoR1	adiponectin receptor 1
AdipoR2	adiponectin receptor 2
AdipoRon	adiponectin receptor agonist (AdipoRon)
AMP	adenosine monophosphate
AMPK	AMP-activated protein kinase
COX-2	cyclooxygenase 2
DNA	deoxyribonucleic acid
GCF	gingival crevicular fluid
HMW	high molecular weight
IL-(1-18)	interleukin (1–18)
*LEPR*	leptin receptor gene
LPS	lipopolysaccharide
NF-κB	nuclear factor kappa B
NSPT	non-surgical periodontal therapy
OPG	osteoprotegerin
PDL	periodontal ligament
RA	rheumatoid arthritis
RANKL	receptor activator of nuclear factor kappa B ligand
TNF-α	tumour necrosis factor alpha
